# Effect of different axial speed patterns on cyclic fatigue resistance of rotary nickel-titanium instruments

**DOI:** 10.1186/s12903-022-02639-8

**Published:** 2022-12-18

**Authors:** Myint Thu, Arata Ebihara, Keiichiro Maki, Miki Nishijo, Shunsuke Kimura, Taro Nakatsukasa, Moe Sandar Kyaw, Takashi Okiji

**Affiliations:** Division of Oral Health Sciences, Department of Pulp Biology and Endodontics, Graduate School of Medical and Dental Sciences, Tokyo Medical and Dental University, 5-45, Yushima 1-chome, Bunkyo-ku, Tokyo, 113-8549 Japan

**Keywords:** Dynamic cyclic fatigue resistance, Nickel-titanium rotary instruments, Pecking speed, Root canal instrumentation

## Abstract

**Background:**

To evaluate the effect of pecking motions with faster upward speed on the dynamic cyclic fatigue resistance of nickel-titanium rotary instruments with different metallurgy.

**Methods:**

Forty each of ProTaper Universal F3 (PTU) and ProTaper Gold F3 (PTG) instruments (size #30/.09) were equally divided into four groups. The test was performed using an 18-mm-long stainless steel artificial canal with a 5-mm radius of curvature, a 45° canal curvature and a 2-mm canal diameter. A downward speed of 100 mm/min was employed, while the upward speed was set at 100, 150, 200 or 300 mm/min. Time to failure (T*f*), number of cycles to failure (N*f*) and number of pecking motions to failure (N*p*) were recorded. Statistical analysis was performed using Kruskal Wallis and Mann–Whitney U tests for T*f*, N*f,* and N*p* (*α* = 0.05).

**Results:**

The 100/300 mm/min group showed significantly higher N*p* values than the 100/100 mm/min group (*p* < 0.05), whereas there were no significant differences in T*f* and N*f* among the tested speed groups (*p* < 0.05) in either PTU or PTG. PTG exhibited significantly higher T*f*, N*f,* and N*p* than PTU (*p* < 0.05).

**Conclusions:**

Under the tested conditions, the fastest upward speed group showed significantly higher cyclic fatigue resistance, as demonstrated by larger N*p*, than the same speed group. PTG had significantly higher cyclic fatigue resistance than PTU in all groups.

**Supplementary Information:**

The online version contains supplementary material available at 10.1186/s12903-022-02639-8.

## Background

Root canal cleaning and shaping with endodontic instruments is an essential phase of root canal treatment to create a space for three-dimensional disinfection and obturation [1]. Use of endodontic nickel-titanium (NiTi) instruments made from nitinol wire was pioneered by Walia et al. in 1988 [2]. In contrast with traditional stainless steel files, motorized NiTi rotary instruments are considered to facilitate root canal preparation with fewer procedural errors while maintaining the original root canal geometry [3–5].

However, separation of NiTi instruments remains one of the main complications in clinical endodontics despite advancements in metallurgy and design, which might occur at different stress/strain levels, either in the presence or absence of an obvious plastic deformation mark next to the fracture area [6–8]. Most instrument fractures arise due to cyclic fatigue, which is known to occur when the repeated compression/tension stresses gather through the curvature of the rotating instrument [10].

Laboratory cyclic fatigue tests are conducted in either static or dynamic mode with major differences in the axial motion [11–14]. The dynamic cyclic fatigue test simulates clinical conditions as it incorporates an axial motion (pecking motion), and is known to extend the fatigue resistance of the instruments by distributing the accumulated stress along the instrument [11–14].

The existing literature indicates that a same speed is widely adopted for the pecking motion in dynamic cyclic fatigue tests [11, 13–18]. However, it is possible that the withdrawal speed of the instrument during clinical instrumentation is faster than the insertion speed as a result of resistance release [12]. The manipulation style of the instruments during canal instrumentation is important as a preventive measure for intracanal instrument fracture [19]. It is therefore important to know how pecking motions with different axial speed patterns (such as same speed versus faster upward speed) affect the cyclic fatigue resistance of the instruments.

To the best of the authors' knowledge, there is no study investigating the effects of pecking motions with different axial speed patterns on the fatigue-reducing effect of the instruments. Hence, the aim of the present study was to examine the effect of different axial speed patterns on the dynamic cyclic fatigue resistance, using two NiTi rotary instruments with the same geometry but made of different NiTi alloys. The null hypothesis tested was that there would be no difference in time to failure (T*f*), number of cycles to failure (N*f*), and number of pecking motions to failure (N*p*) among the tested speed groups in each instrument system and also between the two instrument systems.

## Materials and methods

G*Power (Version 3.1.9.6 for Mac OS X, Heine Heinrich Universität, Düsseldorf, Germany) was used for sample size estimation with an effect size of 0.75, α error of 0.05, and power of 0.95 in accordance with a previous study [12], and a total of 80 samples (n = 10, per group) was indicated.

Forty instruments each of ProTaper Universal F3 (PTU; #30/0.09; Dentsply Maillefer, Ballaigues, Switzerland) and ProTaper Gold F3 (PTG; #30/0.09; Dentsply Maillefer, Ballaigues, Switzerland) with a length of 25 mm were tested in this study. Inspection for observable production defects was performed at 25 × magnification under a dental operating microscope (MANI Dental Microscope Z; MANI, Utsunomiya, Japan).

**Fig. 1:**
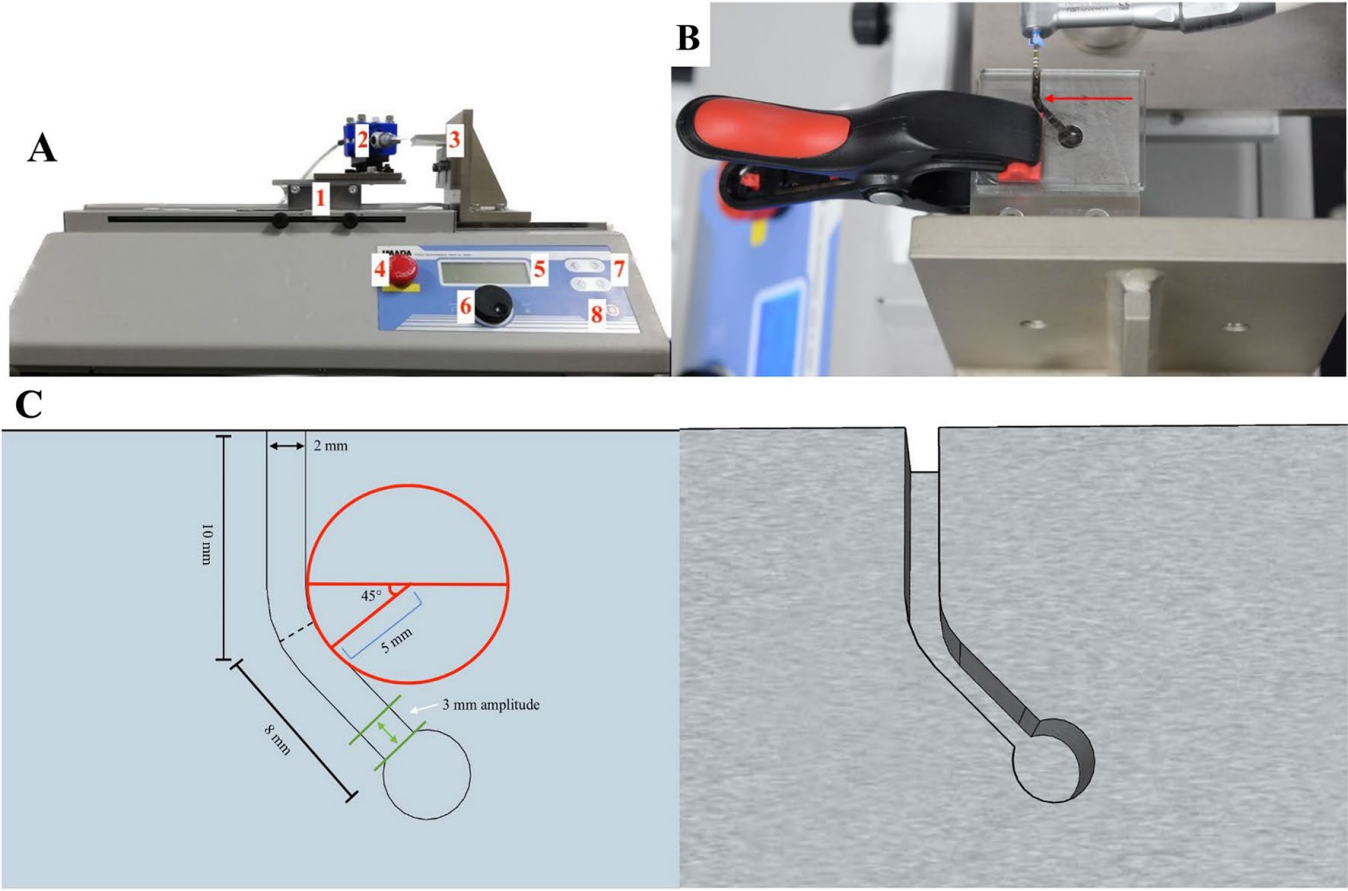
**a** Custom-made cyclic fatigue testing device. (1) movable test stand, (2) handpiece, (3) attached stainless steel artificial canal, (4) emergency stop button, (5) indicator, (6) setting button, (7) start and manual speed adjustment buttons and (8) stop button. **b** Stainless steel artificial canal (arrow). **c** Schematic diagrams of stainless steel artificial canal

A dynamic cyclic fatigue test was performed using a customized testing device as previously described [12]. Briefly, the testing device contained a test stand with a movable stage (MH2-500N; IMADA, Toyohashi, Japan), where the handpiece of an X-Smart Plus motor (Dentsply Maillefer) was mounted (Fig. 1a). An 18-mm-long stainless steel artificial canal with a 45° angle of curvature, 5-mm radius of curvature, and 2-mm canal diameter was used (Fig. 1b, c). The downward and upward speeds of pecking motion in the same speed group were set at 100 mm/min (100/100 mm/min group) and served as a control. In the faster upward speed groups, the upward speed was set at 150, 200 and 300 mm/min (100/150, 100/200 and 100/300 mm/min groups, respectively). Each instrument type was equally divided into four groups (n = 10 each). The center of the curvature of the canal was 8 mm from the apex. The instruments were fixed at 15 mm in the artificial canal and then extended to the working length of 18 mm with 3 mm amplitude to simulate the pecking motion.

To reduce the frictional heat produced by the contact between the instruments and the artificial canal wall, silicone oil (KF-96-100CS; Shin-Etsu Chemical, Tokyo, Japan) was sprayed on each instrument before testing [12, 20]. A transparent glass plate was placed over the artificial canal. As recommended by the manufacturer, the instruments were operated at 300 rpm and 3 Ncm [21]. Until failure was noticed visually and audibly, T*f* was recorded in seconds using a stopwatch (Time Keeper; Seiko, Tokyo, Japan). Videos were simultaneously documented with a digital single lens reflex camera (Nikon D5300; Nikon Imaging, Tokyo, Japan) throughout the experiment and T*f* was reaffirmed with the recorded videos assessed by three examiners, including two blind examiners. The N*f* was obtained by multiplying rpm and T*f* (min) [12]. The N*p* was attained by the computerized data shown in the indicator on the test stand where videos were also taken simultaneously to ensure the accuracy of N*p* and fracture occurrence. The experiment was conducted at room temperature (23 ± 1.5 °C). The temperature was maintained by the air conditioning system, and an indoor digital thermometer/hygrometer was used to indicate the room temperature. Under a dental operating microscope, the fragment lengths were measured using a digital caliper (Mitutoyo, Kawasaki, Japan). Fractographic analysis of the fracture surface was performed using a scanning electron microscope (SEM; JSM-7900F Schottky Field Emission Scanning Electron Microscope, JEOL, Tokyo, Japan) at an acceleration voltage of 5 kV (n = 4 each).

The Kolmogorov–Smirnov test for normal distribution and Levene’s test for homogeneity of variance were used. As T*f*, N*f*, and N*p* were non-normally distributed, intergroup comparisons of these values were performed using the Kruskal Wallis test followed by the Mann–Whitney U test with Bonferroni correction*.* Differences in T*f*, N*f*, and N*p* between PTG and PTU were analyzed using the Mann–Whitney U test. Fragment length values showed normal distribution and homogeneous variance and thus were analyzed using two-way analysis of variance. All the statistical analysis was performed using SPSS v27 (IBM Corp, Armonk, NY) at a significance level of 0.05.

## Results

The mean value of T*f* obtained by 3 examiners was presented in Additional file 1: Supplemental Table 1. The median and interquartile ranges of T*f* and N*f* are presented in Tables 1 and 2, respectively. There were no significant differences in T*f* and N*f* among groups with a different upward speed in either PTU or PTG (*p* > 0.05). PTG showed significantly higher T*f* and N*f* than PTU in all tested groups (*p* < 0.05).

**Table 1:** Median and interquartile range (IQR) of time to failure (T*f*) of instruments (seconds)

Files	T*f*							
	100/100		100/150		100/200		100/300	
	Median	IQR	Median	IQR	Median	IQR	Median	IQR
PTU	175.76^A,a^	172.99–203.90	188.04^A,a^	171.81–219.32	198.60^A,a^	190.36–211.14	198.68^A,a^	181.68–213.92
PTG	425.17^A,b^	402.52–440.41	501.24^A,b^	407.74–511.28	455.88^A,b^	370.27–494.80	440.54^A,b^	414.05–473.02

Different superscript uppercase letters (A) indicate significant differences in each row (*p* < 0.05), and different superscript lowercase letters indicate significant differences in each column (*p* < 0.05)

**Table 2:** Median and interquartile range (IQR) of number of cycles to failure (N*f*) of instruments

Files	N*f*							
	100/100		100/150		100/200		100/300	
	Median	IQR	Median	IQR	Median	IQR	Median	IQR
PTU	878.82^A,a^	864.95–1019.49	940.22^A,a^	859.06–1096.60	993.02^A,a^	951.82–1055.69	993.42^A,a^	908.41–1069.61
PTG	2125.85^A,b^	2012.61–2202.05	2405.60^A,b^	2020.34–2540.24	2279.40^A,b^	1851.36–2474.01	2201.25^A,b^	2070.25–2365.11

Different superscript uppercase letters (A) indicate significant differences in each row (*p* < 0.05), and different superscript lowercase letters indicate significant differences in each column (*p* < 0.05)

The 100/300 mm/min group showed significantly higher N*p* compared with the 100/100 mm/min group in both PTU and PTG (*p* < 0.05) (Table 3). There were no significant differences in N*p* among the faster upward speed groups in either of the instrument systems (*p* > 0.05). PTG demonstrated a significantly higher N*p* than PTU in all tested speed groups (*p* < 0.05).

**Table 3:** Median and interquartile range (IQR) of number of pecking motions to failure (N*p*) of instruments

Files	N*p*							
	100/100		100/150		100/200		100/300	
	Median	IQR	Median	IQR	Median	IQR	Median	IQR
PTU	48^A,a^	42.25–50.75	58.50^AC,a^	51–63.62	63^AC,a^	61.25–70.25	109.50^BC,a^	106.37–125
PTG	70.75^A,b^	64.87–78.50	140^AC,b^	124.62–152.37	144^AC,b^	118.50–160	151.50^BC,b^	144.25–171.12

Different superscript uppercase letters indicate significant differences in each row (*p* < .005), and different superscript lowercase letters indicate significant differences in each column (*p* < 0.05)

There was no significant difference in fragment length among the tested groups or between PTU and PTG (*p* > 0.05; Table 4), and 57.5% of PTU instruments and 62.5% of PTG instruments fractured during downward movement.

**Table 4:** Mean and standard deviation of fragment length (FL) of instruments (mm)

Files	FL			
	100/100	100/150	100/200	100/300
PTU	7.68 (0.37)^A,a^	7.89 (0.50)^A,a^	7.57 (0. 53)^A,a^	7.71 (0.49)^A,a^
PTG	7.96 (0.60)^A,a^	8.29 (0.72)^A,a^	8.18 (0.71)^A,a^	7.74 (0.72)^A,a^

Different superscript uppercase letters (A) indicate significant differences in each row (*P* < .05), and different superscript lowercase letters indicate significant difference in each column (*P* < .05)

**Fig. 2:**
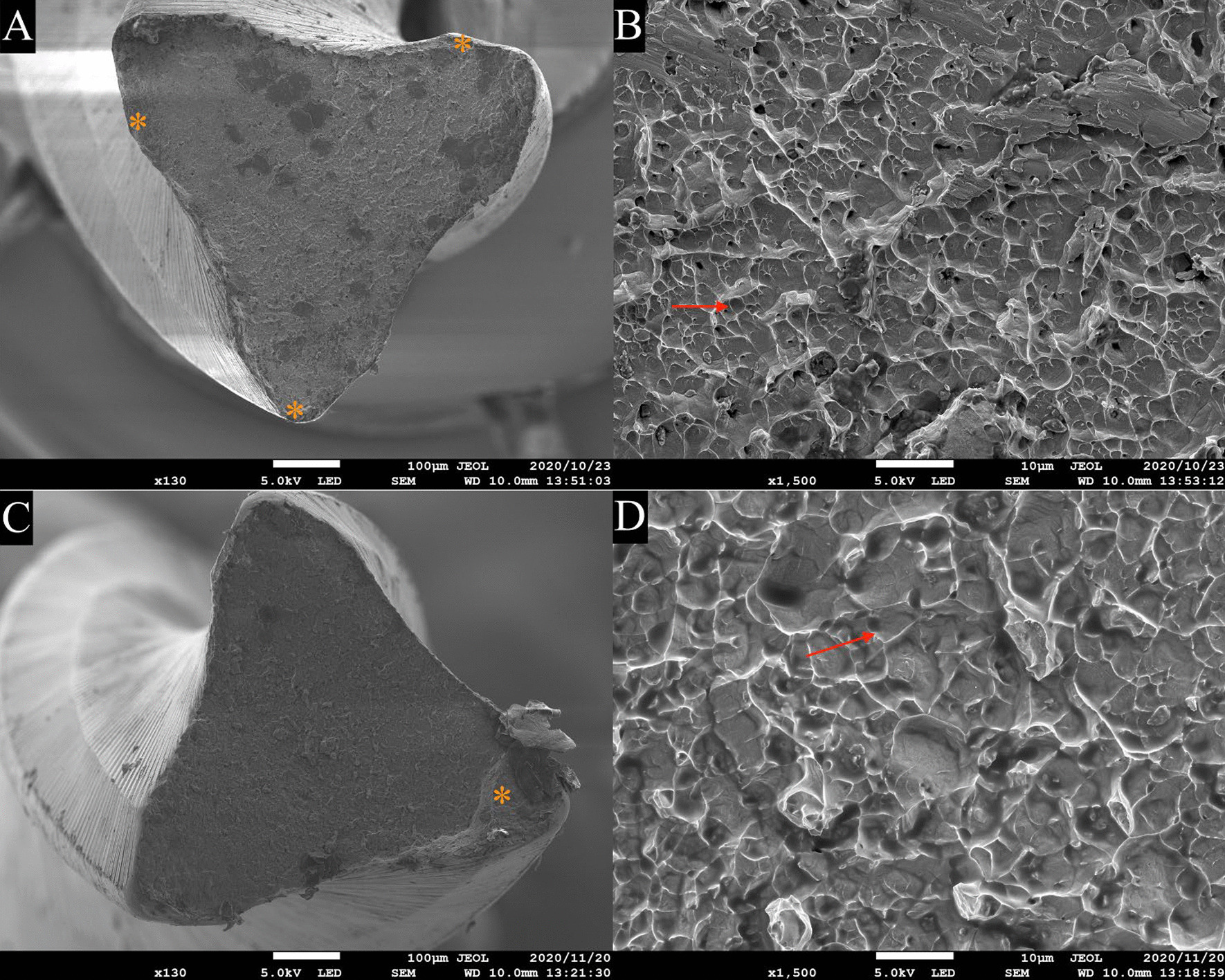
Representative SEM images of the fractured surface of the broken fragment of ProTaper Gold F3 (**a, b**) and ProTaper Universal F3 (**c, d**) after dynamic cyclic fatigue test. *Asterisk*, crack initiating area; red arrows, dimples

Representative SEM images (Fig. 2) show typical features of cyclic fatigue failure, including crack initiating areas and dimples.

## Discussion

The effect of pecking motions with the same upwards and downwards speeds and faster upward speeds on the dynamic cyclic fatigue resistance of instruments with similar designs and sizes was investigated in the present study. Despite T*f* and N*f* exhibited non-significant differences, there were significant differences in N*p* between the 100/100 mm/min group and the 100/300 mm/min group in each instrument system. Thus, the first hypothesis, that there would be no difference in T*f*, N*f*, and N*p* among the tested speed groups in each instrument system, was partly rejected. The second hypothesis, that there would be no difference in T*f*, N*f*, and N*p* between the two instrument systems, was also rejected because significant differences in T*f*, N*f*, and N*p* were observed between the two instrument systems in all tested groups.

To the best of our knowledge, the present study is the first study investigating the effect of different axial speed patterns on the fatigue reduction during NiTi rotary instrumentation. The automated cyclic fatigue testing device was used to produce constant speed and movement with stroke motion. No consensus on clinical insertion and withdrawal speeds is available, and the practical withdrawal speeds may vary from clinician to clinician. Thus, we used the different faster upward speeds at certain values to understand the behaviors of the tested instruments relating to cyclic fatigue resistance, in comparison with same speed. As it is essential to retain the geometric features of the canal to standardize the experimental condition, we used the stainless steel artificial canal. It has been reported that the instruments’ size and cross-sectional design are among the influencing factors on cyclic fatigue resistance [22, 23]. To eliminate such variables, PTU and PTG instruments with identical geometrical designs and sizes [24] were selected in the present study.

This study employed N*p* as a factor that influences dynamic cyclic fatigue resistance [25], in addition to T*f* and N*f*, which are widely used to evaluate cyclic fatigue resistance [11–18, 26–29]. N*p *is also considered an important variable that can help estimate how many times a rotating instrument can pass through the highest flexure area by resisting the stresses before breakage. Moreover, groups with different upward speeds will exhibit different N*p* values even when the duration of the instrumentation, and thus T*f* and N*f*, is similar among groups. For these reasons, N*p *was employed to evaluate the dynamic cyclic fatigue resistance in the present study.

It has been reported that the faster pecking motions with the same upwards and downwards speeds significantly improved the fatigue life of the instruments [25]. Besides, our previous study showed that a dynamic cyclic fatigue test with faster upward speed significantly extended the fatigue resistance of the continuous rotary instruments in contrast to the static cyclic fatigue test [12]. In spite of the non-significant differences in T*f* and N*f* among the tested speed groups for each instrument system, the 100/300 mm/min group exhibited significantly greater N*p* in both PTU and PTG compared with the same speed group in the present study. These outcomes are inconsistent with the results reported by a previous study, which may be due to the application of different methodologies [25].

The finding of the present study indicates that the instruments in the fastest upward speed group with the largest speed difference between downward and upward movements travelled significantly more frequently in and out of the artificial curved canal. Thus, it seems reasonable to suppose that the instruments in the 100/300 mm/min group withstood the repeated tensile and compressive stresses loaded into a shorter cycle, indicating higher resistance to failure than the same speed group. This can be explained by the mechanism by which the fastest upward speed enhanced the quickest release of the stress accumulated in the rotating instrument before the next downward movement.

PTG instruments showed significantly higher T*f*, N*f*, and N*p* than those of PTU instruments in all tested groups. These results (T*f* and N*f)* are in agreement with several previous studies [21, 29–31]. The difference between PTG and PTU instruments may be attributable to the metallurgy of the alloys. A previous study has reported that PTG exhibits two-phase transformation behavior (austenite – R-phase – martensite), and the austenite finish temperature is 50.1 ± 1.7 °C for PTG and 21.2 ± 1.9 °C for PTU [21]. Hence, it can be assumed that PTG and PTU are mainly composed of the martensite/R-phase and austenite phases, respectively, at both room and body temperature [21]. Thus, the experimental room temperature used in this study may have had no significant impact despite the fact that the modulus of elasticity of PTG might be greater at the body temperature. PTG is reported to be more flexible and suffer less stress, which increases cyclic fatigue resistance compared to PTU with comparable geometric designs and sizes when a similar strain is applied [21], which is consistent with our findings. The effect of the different handle lengths, 13 mm in PTU and 11 mm in PTG, on the cyclic fatigue resistance of the instruments remains unexamined yet. However, this may not have significantly affected the results of the present study.

Non-significant differences in the fragment length among the tested speed groups and between the instrument systems in the present study may imply similar bending movements of the instruments [12]. The instruments fractured during both downward and upward movements in the present study, suggesting that the instruments experienced stress at the curvature during both movements as long as they have contact areas with the canal walls. The lower fracture rate during the upward movement could be related to the immediate stress-releasing mechanism that just starts beyond the curvature, where the instrument size and the canal size are indirectly proportional, and the contact area decreases in size during the upward movement. Additionally, it seems reasonable to suppose that the instruments had lesser compression and tension cycles in the same period of time when the upward speed is increased.

In the present study, the cyclic fatigue resistance against the stresses produced along the axis of the artificial curved canal was measured by N*p* in the same axis, which can be a reliable indicator for cyclic fatigue resistance because it is in accordance with the defined mechanism of the cyclic fatigue fracture [10–18]. However, one limitation of this study is that only two types of instruments were examined. Moreover, no universal consensus for cyclic fatigue resistance testing is accessible so far, thus, the different methodologies have widely been adopted [10, 32]. Pecking speeds have an effect on torque and screw-in force of the instruments [33], suggesting that pecking speed setting is a factor that poses methodological consideration. Besides, the lack of clinical adaptability of the artificial stainless steel canal is another limitation of this study. Because various factors including its geometric features and surface roughness, real-time torque, screw-in force, and reaction torque in the stainless steel artificial canal may differ from the natural root canal. Hence, the feasible clinical impact of faster upward speeds on the cyclic fatigue resistance should be further investigated attentively, incorporating the effect of upward pecking speeds on those factors. It should also be noted that the variations in geometric characteristics may alter the instruments' resistance to cycle fatigue [34]. Therefore, further research should be done to validate the findings of this study. Additionally, it has been noted that the cyclic fatigue resistance of NiTi instruments considerably decreased when used repeatedly in clinical settings [35, 36]. Hence, further study is suggested to determine if the different axial speed patterns improve the cyclic fatigue resistance of repeatedly used instruments.

## Conclusions

Within the limitations of the present study, the fastest upward speed group with the largest speed difference displayed significantly higher dynamic cyclic fatigue resistance, as demonstrated by the larger N*p*, than the same speed group*.* PTG exhibited significantly higher cyclic fatigue resistance than PTU in all groups.

## Supplementary Information

**Additional file 1: Supplemental Table 1.** Mean value of Tf (seconds) obtained by 3 examiners.

## Data Availability

The datasets generated during and/or analyzed during the current study are available from the corresponding author on reasonable request.

## Abbreviations

T*f*: Time to failure
N*f*: Number of cycles to failure
N*p*: Number of pecking motions to failure
PTG: ProTaper Gold
PTU: ProTaper Universal
NiTi: Nickel-titanium
