# Supplementary material for: Effect of different axial speed patterns on cyclic fatigue resistance of rotary nickel-titanium instruments
Source: BMC Oral Health. 2022 Dec 18;22:617. doi: 10.1186/s12903-022-02639-8 (PMC9761994; doi:10.1186/s12903-022-02639-8)
Supplement: Supplementary file 1 — Additional file 1: Supplemental Table 1. Mean value of Tf (seconds) obtained by 3 examiners. [file 12903_2022_2639_MOESM1_ESM.docx]

Supplemental Table 1 Mean value of T*f* (seconds) obtained by 3 examiners

| Files | T*f* | | | | | | |
| --- | --- | --- | --- | --- | --- | --- | --- |
|  | 100/100 |  | 100/150 |  | 100/200 |  | 100/300 |
| PTU | 183.36 |  | 197.49 |  | 201.17 |  | 196.78 |
| PTG | 425.61 |  | 474.07 |  | 436.18 |  | 443.71 |
